# Editorial: Diagnosis of zoonoses: Relevance of BSL3 and BSL4 facilities

**DOI:** 10.3389/fcimb.2022.1052082

**Published:** 2022-10-17

**Authors:** Kavita Berger, Anna Rosa Garbuglia

**Affiliations:** ^1^ Board on Life Sciences, National Academies of Sciences, Engineering, and Medicine, Washington, DC, United States; ^2^ Laboratory of Virology, National Institute for Infectious Diseases (INMI) National Institute of Infectious Diseases “L. Spallanzani”, Rome, Italy

**Keywords:** biosafety, biosecurity, pandemic, BLS 3 and BLS 4 laboratories, DURC

The health, societal, and economic devastation caused by the emergence, re-emergence, or deliberate release of infectious diseases has led to numerous international efforts in building national, regional, and international capacity to counter natural, accidental, and deliberate biological incidents. Biosafety and biosecurity are critical to protecting research and public health scientists as they conduct core activities to prevent, detect, and respond to infectious disease outbreaks. International efforts such as the Global Health Security Agenda seek to equip countries with the resources and tools needed to assess their existing capacities, including national capacities in biosafety and biosecurity, and identify pathways for addressing gaps in needed capacities.

The COVID-19 pandemic has led to renewed focus on improving existing capacities, such as pathogen detection and biorisk management, and building new capacities, such as local capacity to develop vaccines. Previous investments in pathogen detection technologies for early identification of potential outbreaks of international concern and characterization of pathogens causing outbreaks are continuing as countries seek to improve their abilities to detect new infections quickly. New mobile technologies were developed and used in several countries to track and help to contain the spread of SARS-CoV-2 infections. Further, a new method for surveillance of virus in wastewater has contributed to public health efforts to monitor incidence of SARS-CoV-2 and now is being used to detect and monitor the spread of monkeypox (Kreidler, 2022). Studies involving specialized surveillance systems to monitor pathogen incidence and prevalence and identification of predominant variants for early prediction of epidemic occurrence of pathogens may be conducted in high containment laboratories, in which biosafety and biosecurity practices are implemented. Appropriate infrastructure and standardized protocols are needed to combat epidemics effectively (Nacoti et al.). Questions and concerns about laboratory biosafety and biosecurity (collectively referred to as biorisk management) persist and lead to new research efforts focused on understanding sources of human error, rather than engineering errors, which have been the traditional focus of biosafety and biosecurity discourse. Information from these studies is anticipated to inform laboratory biosafety and biosecurity risk assessments, and prevention and mitigation measures. A relatively new development resulting from the COVID-19 pandemic is Africa’s interest in and commitment toward developing local capacity for vaccine manufacturing over the next two decades (Africa CDC, 2022). This interest in building international research and development capacity for vaccines against emerging and re-emerging infectious disease is but one example of innovation resulting from the pandemic. The mobile technologies and wastewater surveillance, use of an experimental vaccine platform to combat an emerging infectious disease, and numerous research studies involving non-life scientists to help characterize the virus and pandemic dynamics of COVID-19 all are examples of new innovations in science and technology to help address the ongoing pandemic.

Also during the past few years, concerns about dual use potential of certain SARS-CoV-2 research was raised by biorisk management and biosecurity policy experts, (Musunuri, et al. 2021; Sandbrink, et al. 2021) and has resulted in new look at U.S. policies on “gain-of-function” research (Kaiser, 2022). Beyond the dual use issues, new concerns about the volume of information with variable accuracy has elicited significant concern.

In this Research Topic, publications describe critical, intersecting scientific, security, and health issues associated with investments to prevent, detect, and respond to emerging infectious diseases. Some papers highlight the lack of definition of high containment biological laboratories (HCBLs) in many rules and regulations for their management. HCBLs have a fundamental role in ensuring the safety of researchers working to characterize human and animal pathogens, and conducting preclinical studies for the development of vaccines and new diagnostic tests. The biosafety guidelines promulgated by the World Health Organization (WHO) and the U.S. Centers for Disease Control and Prevention (CDC) are widely accepted by institutions in many countries. However, compliance with and adherence to these guidelines are not mandatory. Authors suggest that achieving standard oversight at an international level and harmonizing the guidelines for working with pathogens are necessary. Although many laboratories are certified under ISO, 9001: 2015 Quality Management Systems, comparatively few are certified under ISO, 35001: Biorisk Management for Laboratory because it is optional. Even the programs on the organization of training (training programs) vary greatly depending on the structure and expertise of the trainer and previous experiences of researches (Moritz et al., 2021). Because of different laboratory designs and resulting operating procedures (SOPS), training from one facility often is not necessarily applicable to another. Biosecurity-specific policies are variable: no common or consistent policies on dual use search of concern and “gain-of-function” exist which leads to ambiguity in their interpretation and variability in use (Yeh et al., 2021).

The articles included in this Research Topic provide insight into the beneficial uses of high containment facilities, particularly in assessing, monitoring, and characterizing emerging infectious diseases. This special issue also highlights current challenges in ensuring biosafety and biosecurity concerns are addressed to maximize the benefits of the scientific activities conducted within these facilities. Many of these issues are expected to remain as new outbreaks emerge and learning from current and past examples to improve capabilities for prevention, detection, and response may be needed.

## Author contributions

All authors listed have made a substantial, direct, and intellectual contribution to the work and approved it for publication.

## Conflict of interest

The authors declare that the research was conducted in the absence of any commercial or financial relationships that could be construed as a potential conflict of interest.

## Publisher’s note

All claims expressed in this article are solely those of the authors and do not necessarily represent those of their affiliated organizations, or those of the publisher, the editors and the reviewers. Any product that may be evaluated in this article, or claim that may be made by its manufacturer, is not guaranteed or endorsed by the publisher.
